# White-Light Photosensors Based on Ag Nanoparticle-Reduced Graphene Oxide Hybrid Materials

**DOI:** 10.3390/mi9120655

**Published:** 2018-12-11

**Authors:** Wei-Chen Tu, Xiang-Sheng Liu, Shih-Lun Chen, Ming-Yi Lin, Wu-Yih Uen, Yu-Cheng Chen, Yu-Chiang Chao

**Affiliations:** 1 Department of Electronic Engineering, Chung Yuan Christian University, Taoyuan 32023, Taiwan; a2001751@gmail.com (X.-S.L.); chrischen@cycu.edu.tw (S.-L.C.); wuyih@cycu.edu.tw (W.-Y.U.); 2 Department of Electrical Engineering, National United University, Miaoli 36003, Taiwan; mylin@nuu.edu.tw; 3 School of Electrical and Electronic Engineering, Nanyang Technological University, Singapore 639798, Singapore; yucchen@ntu.edu.sg; 4 Department of Physics, National Taiwan Normal University, Taipei 10610, Taiwan; ycchao@ntnu.edu.tw

**Keywords:** photosensor, reduced graphene oxide, Ag nanoparticles, solution process, finite-difference time-domain

## Abstract

The unique and outstanding electrical and optical properties of graphene make it a potential material to be used in the construction of high-performance photosensors. However, the fabrication process of a graphene photosensor is usually complicated and the size of the device also is restricted to micrometer scale. In this work, we report large-area photosensors based on reduced graphene oxide (rGO) implemented with Ag nanoparticles (AgNPs) via a simple and cost-effective method. To further optimize the performance of photosensors, the absorbance and distribution of the electrical field intensity of graphene with AgNPs was simulated using the finite-difference time-domain (FDTD) method through use of the surface plasmon resonance effect. Based on the simulated results, we constructed photosensors using rGO with 60–80 nm AgNPs and analyzed the characteristics at room temperature under white-light illumination for outdoor environment applications. The on/off ratio of the photosensor with AgNPs was improved from 1.166 to 9.699 at the bias voltage of −1.5 V, which was compared as a sample without AgNPs. The proposed photosensor affords a new strategy to construct cost-effective and large-area graphene films which raises opportunities in the field of next-generation optoelectronic devices operated in an outdoor environment.

## 1. Introduction

Two-dimensional material graphene with unique properties distinguished from bulk materials has been one of the most celebrated inventions [1,2] in the applications of photosensors and therefore the development of high-efficiency photosensors has become an emerging area of research [3,4,5]. The interesting properties of two-dimensional graphene materials include high flexibility, high mobility, a large surface to volume ratio, broadband absorption which results from its structure, symmetrical conduction/valence bands and the linear dispersion of Dirac massless electrons [6,7,8]. However, photosensors based on single-layer graphene usually exhibit low responsivity and short carrier lifetime due to their zero bandgap. Moreover, graphene-based photosensors are typically fabricated under high-temperature and high-vacuum growth conditions. Besides, single-layer graphene may be broken during the transferring process, therefore the size of graphene flakes and devices is limited, which has become the bottleneck for the future development of graphene [9,10]. In recent years, a solution-processed reduced graphene oxide (rGO) with a tunable bandgap that can range from 1.00 eV to 1.69 eV through modifying its oxygen content has gained much attention, owing to its cost advantage. Consequently, the development of rGO devices and rGO nanocomposites has been widely explored [11,12,13,14,15]. To further improve the performance of graphene-based photosensors, several approaches can be applied. For example, authors formed a vertical built-in field in the graphene channel to trap the photoinduced electrons [16] or fabricated a gated multilayer photodetector, integrated on a photonic waveguide to reduce dark current and increase responsivity [17,18,19,20]. However, these structures were conventionally obtained by using the time-consuming e-beam deposition method or a complicated photolithography process. 

Recently, photosensors with plasmonic structures have been proposed as an effective way to improve the absorption in the active layer which would contribute to the enhanced photocurrent [21,22,23,24]. The physics of surface plasmon resonance lie in the oscillation of conducting electrons at the interface of materials with different permitivities, such as the metal and the non-conducting media. By modifying the shape, structure or surrounding of the metal and the non-conducting media, the characteristics of surface plasmon resonance can be tailored, which is of interest to their potential use in optoelectronic devices [25,26]. Based on this theory, the performance of photosensors can be improved by decorating rGO with plasmonic metal nanoparticles. This not only preserves the outstanding properties of the original material but also provides the improved characteristics. For example, the Ag nanoparticles have a superior ability to enhance the photocurrent through light scattering and the surface plasmon resonance effect [27,28,29]. As a result, graphene with plasmonic nanoparticles can construct a hybrid system that benefits modified and enhanced light absorption [30]. While Ag nanoparticle-graphene nanocomposites have demonstrated enhanced carrier separation, very little research has been performed for the Ag nanoparticle-rGO hybrid system constructed on large-area photosensors and operated under white-light illumination for outdoor environment applications. 

Here, we provide the fabrication method of rGO films on SiO_2_/Si substrates to construct large-area photosensors via a cost-effective solution process. To further optimize the characteristics of rGO films and improve the performance of photosensors, the absorbance and distribution of the electrical field intensity of graphene with different size plasmonic Ag nanoparticles was simulated through the finite-difference time-domain (FDTD) method. Then, 60–80 nm Ag nanoparticles combined with rGO were spin-coated on SiO_2_/Si substrates to form the plasmonic photosensors and the characteristics of the photosensors were measured under white-light illumination using a solar simulator for outdoor environment applications. It was found that the photocurrent of photosensors based on rGO with Ag nanoparticles was enhanced and the on/off ratio was therefore improved from 1.166 to 9.699, comparing the photosensors without Ag nanoparticles. This can be attributed to the effective surface plasmon resonance and light scattering effect. Our fabrication procedure and measured results offer a valuable guide to improving the performance of large-area photosensors or other devices via a simple way which can be applied for future wearable and outdoor devices. 

## 2. Materials and Methods 

**Figure 1:**
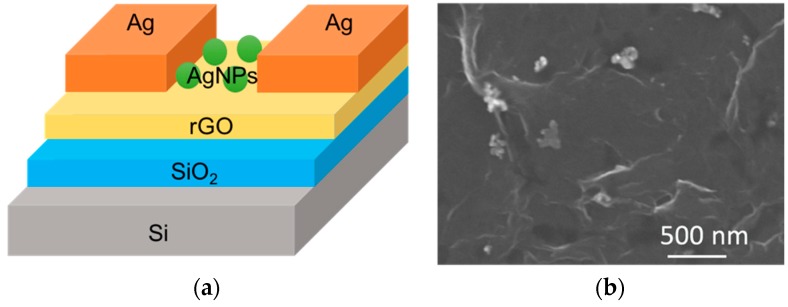
(**a**) Structure of a Ag nanoparticle-reduced graphene oxide (AgNPs-rGO) photosensor. The Ag nanoparticles mixed with reduced graphene oxide (rGO) were spin-coated on SiO_2_/Si to improve the performance of photosensors; (**b**) SEM image of rGO with 60–80 nm Ag nanoparticles (AgNPs).

Figure 1a shows the schematic structure of a solution-processed rGO photosensor on a SiO_2_/Si substrate. First, Si substrates were cleaned using hydrofluoric acid and then rinsed using deionized (DI) water to remove the native oxide on the Si. Then, 300 nm-thick of SiO_2_ was deposited on the Si by the sputtering system. The absorbing layer, rGO, was obtained by reducing the graphite oxide (GO). To synthesize rGO, GO in DI water was sonicated followed by adding hydrobromic acid into the GO colloids. Then the mixtures were refluxed in an oil bath at 110 °C for 24 h. The rGO flakes were obtained after the processes of filtration, washing and desiccation. To construct the active layer of photosensors, rGO flakes in DI water were spin-coated on SiO_2_/Si substrates at a spin rate of 3000 rpm. Finally, a 120 nm Ag film which served as a contact electrode was sputtered on the rGO/SiO_2_/Si. The size of the photosensor was 1.5 cm in width and 1.5 cm in length; the rGO detective channel of the photosensor was 0.2 cm in width and 1.5 cm in length. To further improve the performance of photosensors, 60–80 nm Ag nanoparticles (commercial products produced by Golden Innovation Business Company) mixed with rGO solution were spin-coated on SiO_2_/Si at a spin rate of 3000 rpm. The shape and the diameter of the Ag nanoparticles were spherical and 60–80 nm, respectively. The structure and fabrication processes of the rGO and Ag nanoparticle-rGO photosensor were the same besides the adding of Ag nanoparticles. The SEM image of the rGO with Ag nanoparticles photosensor on the SiO_2_/Si substrate is illustrated in Figure 1b, indicating that the rGO was sheet-like and the rGO flakes can construct a continuous film. Moreover, the plasmonic Ag nanoparticles were successfully coated on the rGO flakes.

## 3. Results and Discussion

**Figure 2:**
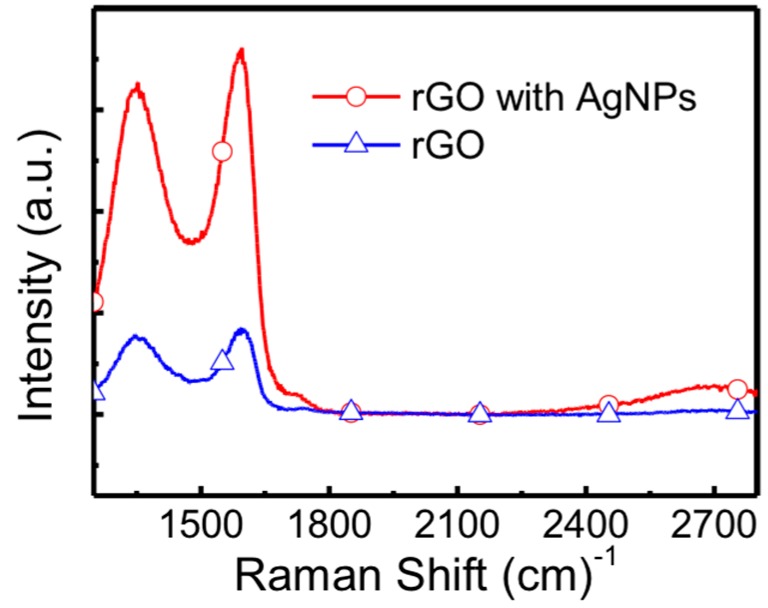
Raman spectra of rGO and Ag nanoparticles-rGO hybrid films. The Raman intensity of rGO with Ag nanoparticles is enhanced owing to the surface plasmon resonance effect.

Raman spectroscopy is a useful and informative technique used to investigate disorder in sp^2^ carbon materials. As shown in Figure 2, characteristic D, G, and 2D peaks of rGO film are observed at 1334, 1591, and 2730 cm^−1^, respectively. The D peak is resulted from the existence of dislocations or vacancies in the graphene and the G peak is originated from the in-phase stretching vibration of symmetric sp^2^ C-C bonds [31,32]. Based on the peaks in the measured Raman spectra, we can confirm that the material we used was rGO. As the Ag nanoparticle combined rGO film is formed, the intensity of Raman spectra exhibits an obvious enhancement due to enhanced Raman scattering induced by the intense local electromagnetic fields of the plasmonic Ag nanoparticles.

**Figure 3:**
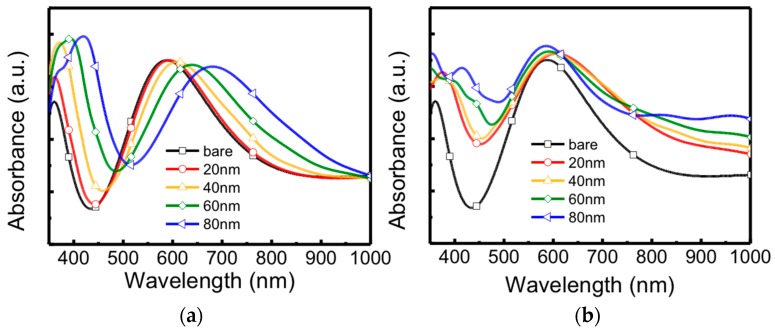
Simulated absorbance spectra of bare graphene and graphene with different size of Ag nanoparticles on (**a**) flat and few-layer graphene/SiO_2_/Si and (**b**) rough and multi-layer graphene/SiO_2_/Si.

The oscillation of carriers generated by the surface plasmon resonance can radiate electromagnetic energy at the same frequency as that of the surface plasmon resonance, which leads to elastic/Rayleigh scattering. Meanwhile, the surface plasmon resonance is dependent on the size and shape of particles as well as the dielectric constant of the surrounding materials [33,34]. To further investigate the surface plasmon resonance effect and the morphology of graphene on the absorbance and distribution of the electric field intensity, we executed simulated absorbance spectra of flat graphene and rough graphene with Ag nanoparticles using the FDTD method. The flat structure was constructed as Ag nanoparticles/flat and few-layer graphene/SiO_2_/Si; the rough structure was designed as Ag nanoparticles/rough and multilayer graphene/SiO_2_/Si. The roughness of graphene was around tens nanometers. Figure 3a,b displays the simulated absorbance spectra of 20 nm, 40 nm, 60 nm and 80 nm Ag nanoparticles on flat graphene/SiO_2_/Si and rough graphene/SiO_2_/Si, respectively. Based on the full Mie equation, for larger nanoparticles (diameter >20 nm), the extinction cross section is dependent on higher-order multipole modes and the extinction characteristics are dominated by quadrupole, octopole absorption and scattering [33]. These higher oscillation modes are related to the particle size, in addition, the maximum absorption may shift to a longer wavelength and the bandwidth will increase with increasing particle size. As displayed in Figure 3a, the absorbance curves of flat graphene with any size of Ag nanoparticles shift toward longer wavelengths, and the larger size of Ag nanoparticles, the larger the shift. In the case of the absorbance spectra of rough graphene with Ag nanoparticles as shown in Figure 3b, the curves also exhibit a shift, however, the shifted wavelengths are not linearly dependent on the particle’s size because the surface plasmon resonance and the light scattering phenomena are more complex. Nevertheless, the frequency of the surface plasmon resonance can be modified and the absorbance of Ag nanoparticles/graphene/SiO_2_/Si is increased in several parts of the wavelength range.

**Figure 4:**
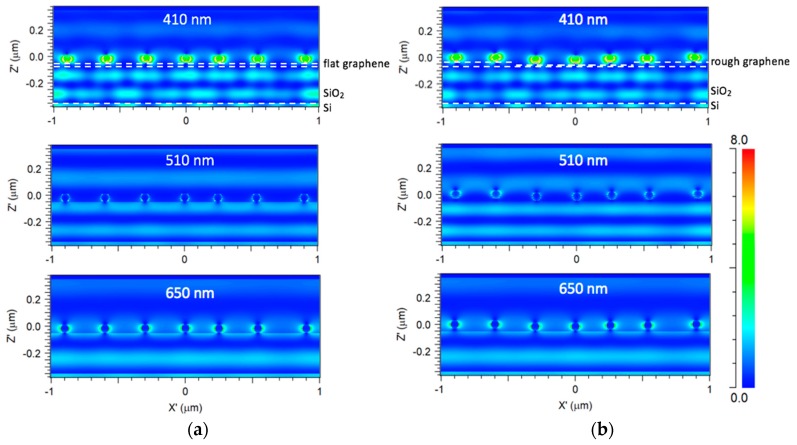
(**a**) Simulated electric field intensity distributions of 60 nm Ag nanoparticles/flat and few-layer graphene/SiO_2_/Si at wavelengths of 410 nm, 510 nm and 650 nm, respectively; (**b**) simulated electric field intensity distributions of 60 nm Ag nanoparticles/rough and multilayer graphene/SiO_2_/Si at wavelengths of 410 nm, 510 nm and 650 nm, respectively.

To explore the effect of graphene morphology on the surface plasmon resonance, the electric field intensity distributions of 60 nm Ag nanoparticles on flat and rough graphene were both simulated. Figure 4a shows the electric field intensity distribution of Ag nanoparticles on flat and few layer graphene/SiO_2_/Si at wavelengths of 410 nm, 510 nm and 650 nm, respectively. The electric field intensities around the Ag nanoparticles at wavelengths 410 nm and 510 nm show the highest and lowest values, respectively, which agrees with the simulated absorption spectra at the wavelengths of 410 nm, 510 nm and 650 nm. Figure 4b exhibits the electric field intensity distribution of Ag nanoparticles on rough and multilayer graphene/SiO_2_/Si at wavelengths of 410 nm, 510 nm and 650 nm, respectively. The roughness of multilayer graphene is about 20 nm. Similarly, the electric field intensity around the Ag nanoparticles at the wavelength of 410 nm is stronger than those of other wavelengths. From these simulated results, it is revealed that the electrical field intensity distributions can be enhanced through the addition of Ag nanoparticles on both flat and rough graphene surfaces.

**Figure 5:**
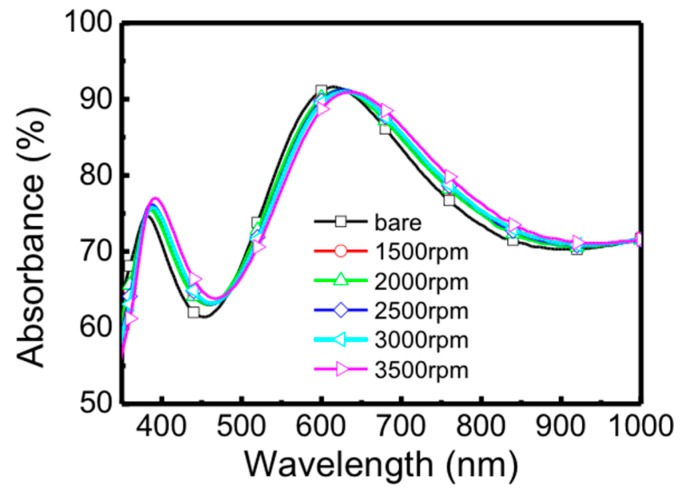
Absorbance spectra of rGO with a spin rate of 3000 rpm and 60–80 nm Ag nanoparticle-rGO with spin rates of 1500 nm, 2000 nm, 2500 nm, 3000 nm and 3500 nm, respectively.

To investigate the difference in the surface plasmon resonance effect between the simulated and experimental results, the optical absorbance spectra of 60–80 nm Ag nanoparticle-rGO on SiO_2_/Si with different spin rates, including 1500 rpm, 2000 rpm, 2500 rpm, 3000 rpm and 3500 rpm were measured through ultraviolet-visible-near infrared (UV-vis-NIR) spectroscopy in a wavelength range from 350 nm to 1000 nm, as shown in Figure 5. When several metal nanoparticles are close to other nanoparticles, the coupling effect between particles becomes very important. By controlling the spin rate of Ag nanoparticles/rGO solution on substrates, the density of Ag nanoparticles and the thickness of rGO can be tuned. Figure 5 displays that the absorbance curves of Ag nanoparticle-rGO with different spin rates is shifted about 10 nm which is beneficial to specific optoelectronic devices.

**Figure 6:**
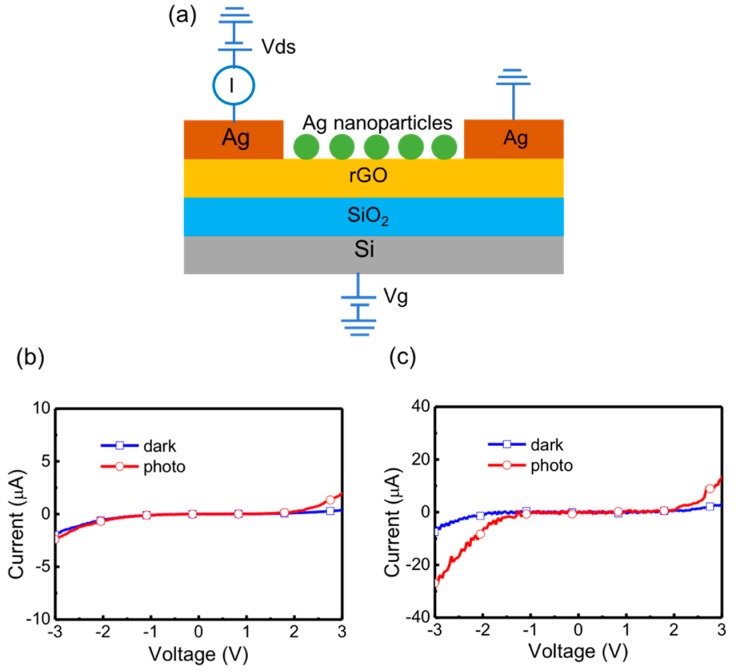
(**a**) Cross-sectional view of the rGO photosensor with electrical connections for current-voltage (I-V) measurement. The substrate is served as a back gate; one of the Ag electrodes sets as drain and the other is the source. Current-voltage curves of (**b**) a rGO photosensor without Ag nanoparticles and (**c**) a rGO photosensor with 60–80 nm Ag nanoparticles.

To design photosensors operated in an outdoor environment, typical current-voltage (I-V) characteristics of rGO and Ag nanoparticle-rGO photosensors under white-light illumination by the light source of an AM 1.5 G solar simulator with power density of 1.03 kW/m^2^ were measured. Figure 6a shows the structure of a rGO-based photosensor with electrical connections to detect the I-V characteristics. The applied voltage of back gate (V_g_) was set as 0 V for low-power applications and one of the Ag electrodes served as a drain and the other electrode as a source. The voltage between the drain and source (V_ds_) was swept from −3 V to +3 V. When the rGO absorbs incident light, excitons (electron-hole pairs) are obtained at the Schottky-like metal-rGO interface. In addition, defects in the rGO film can help dissociate excitons into free carriers and some of them have sufficient energy to overcome the Schottky barrier. In Figure 6b, I-V curves of a rGO photosensor without Ag nanoparticles are displayed. The light and dark currents are distinguishable even at a low bias because the photosensor collects more photocurrent, owing to large absorbing area. I-V characteristics of a photosensor with 60 nm Ag nanoparticles are displayed in Figure 6b. A further improvement of the on/off ratio is achieved by the surface plasmon resonance effect caused by the Ag nanoparticles. At the bias of −1.5 V, the dark current, light current and on/off ratio of the photosensor without Ag nanoparticles was 0.241 μA, 0.281 μA and 1.166, respectively; the dark current, light current and on/off ratio of the photosensor with 60–80 nm Ag nanoparticless was 0.163 μA, 15.809 μA and 9.699, respectively. The responsivities of photosensors without and with Ag nanoparticles at bias voltage of −1.5 V were 0.52 μA/W and 202.53 μA/W, respectively. As incident light with proper frequency illuminates on the plasmonic structure, the oscillation of conducting electrons at the interface of materials arises and the electrical field intensity surrounding the plasmonic structure is increased, leading to the improved photocurrent. As a result, the on/off ratio of rGO with Ag nanoparticles can be enhanced, which is attributed to the plasmon-generated carriers from the AgNPs into the surrounding rGO and the plasmon-enhanced direct carrier excitation of rGO electrons. Additionally, the dark current of the photosensor with Ag nanoparticles shows a higher shunt resistance than that of the photosensor without Ag nanoparticles, resulting in the improved on/off ratio and a lower dark current.

**Figure 7:**
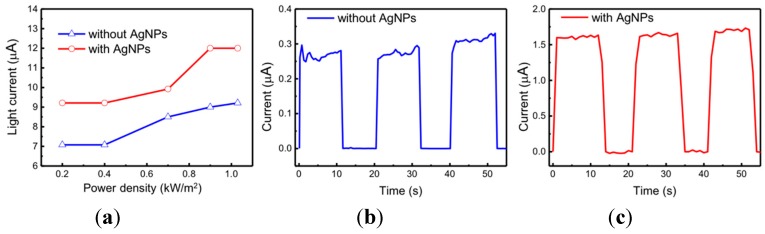
(**a**) Light current as a function of the input light power density under solar simulator illumination for photodetectors with and without Ag nanoparticles at the bias voltage of −2 V. Time-dependent current of photosensors (**b**) without and (**c**) with Ag nanoparticles recorded at the bias voltage 2 V under AM1.5G solar simulator illumination, turning on and off.

In Figure 7a, light currents of photosensors with and without Ag nanoparticles operated at the bias −2 V were measured under different levels of light density illumination. For the photosensor without Ag nanoparticles, more photons were absorbed and they generated more carriers as the intensity of light was increased. For the photosensor with Ag nanoparticles, the photocurrent increased with the increase of light density when the light power density was lower than 0.9 kW/m^2^ and the light current was saturated when the light power density was higher than 0.9 kW/m^2^. This phenomenon can be attributed to the reduced effective trap states and therefore the light current cannot further increase. Figure 7b,c show the time-dependent responses of the photodetectors without and with Ag nanoparticles, respectively. Both devices were operated at the bias voltage of 2 V and the photocurrents were measured by turning on and off the solar simulator which had a power density of 1.03 kW/m^2^. These measured results demonstrate that the photosensors behave well under continuous cycling illumination. To deeply study the response time of photosensors, the rising time was measured as the photocurrent was increased from 10% to 90%. The rising time for the device without and with Ag nanoparticles was 1.0 s and 1.5 s, respectively.

## 4. Conclusions

In summary, large-area rGO photosensors on SiO_2_/Si substrates have been constructed via a cost-effective solution process. To further improve the performance of photosensors, hybrid materials composed of rGO and plasmonic Ag nanoparticles were formed and devices were operated under white-light illumination for outdoor environment applications. In this hybrid system, uniform and continuous rGO films acted as absorbing layer of photosensors and Ag nanoparticles promoted the photocurrent through the surface plasmon resonance effect. As a result, the Ag nanoparticle-rGO photosensors exhibited excellently improved photocurrent and 8-fold increase in the on/off ratio. Besides, the influence of the morphology of graphene on the surface plasmon resonance was discussed using FDTD simulated results. Our study paves a new strategy for the fabrication of an Ag nanoparticle-rGO hybrid system as well as for photosensors, which provides a possible way for the future development of the solution-process and large-area optoelectronic devices used in outdoor environments.
